# Draft Genome Sequences of Pantoea agglomerans, Paenibacillus polymyxa, and *Pseudomonas* sp. Strains, Seed Biogel-Associated Endophytes of Cucumis sativus L. (Cucumber) and *Cucumis melo* L. (Cantaloupe)

**DOI:** 10.1128/MRA.00667-20

**Published:** 2020-08-06

**Authors:** Eman M. Khalaf, Manish N. Raizada

**Affiliations:** a Department of Plant Agriculture, University of Guelph, Guelph, Ontario, Canada; b Department of Microbiology and Immunology, Faculty of Pharmacy, Damanhour University, Damanhour, Egypt; University of Arizona

## Abstract

We report here the draft genome sequences of strains of Pantoea agglomerans (EKM10T, EKM20T, EKM21T, and EKM22T), Paenibacillus polymyxa (EKM10P and EKM11P), and *Pseudomonas* sp. strain EKM23D. These microbes were cultured from fresh seed biogels of Cucumis sativus L. (cucumber) and Cucumis melo L. (cantaloupe). The strains suppress the growth of soilborne fungal/oomycete phytopathogens *in vitro*.

## ANNOUNCEMENT

The seeds of many plants are coated with biogels (mucilage) consisting of polysaccharides that have been shown to promote the growth of animal gut microbes (1), but surprisingly little is known as to whether these biogels host native microbes. The cucurbit family, which includes cucumber and cantaloupe, is well known for its seed biogels. We previously showed that the biogels (mucilage) coating the seeds of different cucurbits host microbes with antimicrobial protective functions (2). This is analogous to defensive antimicrobial biomolecules in the amniotic fluid surrounding developing animal embryos (3, 4). We previously explored the genomes of biogel microbes from wild cucumber (Echinocystis lobata) (5). As such research is novel in the literature, the rationale of this study is to further explore native seed biogel microbe genomes, here from fresh Cucumis sativus L. (cucumber) and Cucumis melo L. (cantaloupe) fruits.

The fruits were grown on commercial farms in Ontario, Canada, and purchased from local markets in the city of Guelph. Following sterilization of the fruit (which was washed with water and soap, sprayed with 70% ethanol, and allowed to dry), the seeds of three fresh fruits (3 replicates) of each crop were aseptically extracted, including their surrounding biogels. The seeds were transferred into Falcon tubes and washed three times with autoclaved double-distilled water (ddH_2_O); then, 100 μl of each wash was streaked onto three agar media (LGI [5], potato dextrose agar [PDA], and Reasoner’s 2A [R2A] agar) (6). From the two crops, here we focus on seven unique bacterial colonies that were isolated and identified using the 16S gene primer pair 799F and 1492R by performing a BLAST search against the NCBI and RDP databases. Three strains originated from the cucumber (*Pantoea* sp. strain EKM10T, *Paenibacillus* sp. strains EKM10P and EKM11P) and four strains from the cantaloupe (*Pantoea* sp. strains EKM20T, EKM21T, and EKM22T and *Pseudomonas* sp. strain EKM23D); they were deposited in GenBank (under the accession numbers MK852351.1, MK852340.1, MK852345.1, MK852348.1, MK852349.1, MK852358.1, MK852361.1, respectively). Several strains of *Pantoea*, *Paenibacillus*, and *Pseudomonas* have been registered/commercialized as crop biocontrol agents (7–10); thus, we previously tested these microbes *in vitro* for their suppressive potential against soilborne pathogens. All strains suppressed oomycetes (Phytophthora capsici and Pythium aphanidermatum), but *Paenibacillus* sp. strains EKM10P and EKM11P exclusively inhibited fungal growth (Fusarium graminearum and Rhizoctonia solani) (2).

Unique strains were preserved and maintained as 50% glycerol stocks at −80°C, which were used for all downstream experiments. From the original glycerol stocks, strains were streaked onto LB agar and incubated overnight at 30°C. Single colonies were selected to inoculate LB broth and were incubated overnight in an orbital shaker at 37°C and 250 rpm; then, genomic DNA was extracted from the harvested bacterial pellets using DNeasy UltraClean microbial kits (12224-50; Qiagen), adjusted to 50 ng/μl. Libraries were prepared using TruSeq DNA Nano library prep kits (KAPA HyperPrep kit, kit code KK8504) and then sequenced using the Illumina NovaSeq 6000 platform. The numbers of output raw reads (2 × 150-bp paired-end format) were 3,149,983 (EKM10T), 3,144,054 (EKM20T), 3,373,222 (EKM21T), 3,170,502 (EKM22T), 2,222,688 (EKM10P), 1,748,173 (EKM11P), and 3,380,471 (EKM23D). The EvoCAT pipeline (Evogene Clustering & Assembly Toolbox) was used to perform *de novo* assembly. The taxonomy was identified using KmerFinder 3.1 (11), with 178× (EKM10T), 172× (EKM20T), 182× (EKM21T), 176× (EKM22T), 91× (EKM10P), 74× (EKM11P), and 105× (EKM23D) sequence coverages when a BLAST search was performed against the top sequence matches of Pantoea agglomerans strain L15 (GenBank accession number NZ_CP034148), Paenibacillus polymyxa strain HY96-2 (NZ_CP025957), and *Pseudomonas* sp. strain TKP (CP006852), with 67.66%, 65.12%, 65.04%, 65.03%, 75.29%, 75.33%, and 14.7% query coverages, respectively. Protein predictions were generated using Prodigal software (12); then, the NCBI nonredundant protein database was queried using BLASTP software to identify the most similar sequences (13). Peptide domains were determined using InterProScan 5.32-71.0 (14). Unless otherwise specified, default settings were used for all software. The characteristics and accession numbers of the genome sequences are presented in Table 1.

**TABLE 1:** Characteristics and accession numbers of sequenced genomes of bacterial endophytes isolated from fresh cucumber and cantaloupe seed biogels

Isolate	Bacterial species^*a*^	Genome size (bp)	No. of clean reads	No. of contigs	*N*_50_ (bp)	No. of genes	G+C content (%)	SRA accession no.	GenBank accession no.
EKM10T	*Pantoea agglomerans*	4,710,579	2,402,254	55	387,357	4,064	55	SRR11051778	JAALEU000000000.1
EKM20T	*Pantoea agglomerans*	4,886,663	2,311,352	69	390,007	4,180	56	SRR11051698	JAALFW000000000.1
EKM21T	*Pantoea agglomerans*	4,874,245	2,445,781	39	420,936	4,170	55	SRR11051719	JAALFV000000000.1
EKM22T	*Pantoea agglomerans*	4,875,195	2,370,526	50	380,273	4,172	55	SRR11051699	JAALFX000000000.1
EKM10P	*Paenibacillus polymyxa*	5,911,943	1,759,810	178	468,459	4,650	52	SRR11038269	JAALET000000000.1
EKM11P	*Paenibacillus polymyxa*	5,909,556	1,420,012	185	670,170	4,663	51	SRR11051718	JAALEV000000000.1
EKM23D	*Pseudomonas* sp.	5,796,589	2,460,061	155	197,140	4,891	60	SRR11051781	JAALFY000000000.1

a Taxonomy of sequenced strains was collected from updated GenBank databases.

All strains possessed genes predicted to encode biomolecules potentially involved in their previously identified antagonistic activities, including bacteriocins (antimicrobial peptides) (15), hydrolytic enzymes (e.g., chitinases [16], proteases [17], pectin/pectate lyase [18]), siderophore-like molecules (19, 20), polyketide synthase (PKS) (8, 21), phenazine biosynthesis protein (PhzF) (19, 22, 23), and metalloenzyme and LuxS/M16 peptidase-like (involved in quorum sensing) (24, 25). Noteworthy is that the *Paenibacillus* strains lacked PhzF but uniquely carried genes implicated in the biosynthesis of β-glucanase (16, 26) and lantibiotics (27). These strains along with *Pseudomonas* sp. strain EKM23D encode cellulases (28, 29) and subtilisin serine protease (30). These findings implicate a diversity of genetic mechanisms underlying the biocontrol activities of seed biogels, a novel microbiome ecological niche.

### Data availability.

This whole-genome shotgun project and raw Illumina reads have been deposited in DDBJ/EMBL/GenBank and the SRA, respectively, under the accession numbers noted in Table 1.

## References

[1] Soukoulis C, Gaiani C, Hoffmann L 2018 Plant seed mucilage as emerging biopolymer in food industry applications. Curr Opin Food Sci 22:28–42. doi:10.1016/j.cofs.2018.01.004.

[2] Khalaf EM, Raizada MN 2019 Cucurbit seed biogels antagonize major plant pathogens. Plant Canada 2019, Guelph, Canada, 7 to 10 July 2019. doi:10.6084/m9.figshare.12554345.v1.

[3] Mao Y, Pierce J, Singh-Varma A, Boyer M, Kohn J, Reems J-A 2019 Processed human amniotic fluid retains its antibacterial activity. J Transl Med 17:68. doi:10.1186/s12967-019-1812-8. 30823930 PMC6397468

[4] Bosso M, Ständker L, Kirchhoff F, Münch J 2018 Exploiting the human peptidome for novel antimicrobial and anticancer agents. Bioorg Med Chem 26:2719–2726. doi:10.1016/j.bmc.2017.10.038. 29122440

[5] Hartmann A, Baldani JI The genus Azospirillum, p 115–140. In Dworkin M, Falkow S, Rosenberg E, Schleifer K-H, Stackebrandt E (ed), The prokaryotes: volume 5: Proteobacteria: alpha and beta subclasses. Springer, New York, NY.

[6] Khalaf EM, Raizada MN 2020 Draft genome sequences of *Acinetobacter* sp. strain EKM10A, *Enterobacter hormaechei* EKM10E, and *Enterobacter hormaechei* EKM11E (Phylum *Proteobacteria*) colonizing the seed surface biogel of *Echinocystis lobata* (wild cucumber). Microbiol Resour Announc 9:e00184-20. doi:10.1128/MRA.00184-20. 32409531 PMC7225530

[7] Smits THM, Duffy B, Blom J, Ishimaru CA, Stockwell VO 2019 Pantocin A, a peptide-derived antibiotic involved in biological control by plant-associated *Pantoea* species. Arch Microbiol 201:713–722. doi:10.1007/s00203-019-01647-7. 30868174

[8] Olishevska S, Nickzad A, Déziel E 2019 *Bacillus* and *Paenibacillus* secreted polyketides and peptides involved in controlling human and plant pathogens. Appl Microbiol Biotechnol 103:1189–1215. doi:10.1007/s00253-018-9541-0. 30603850

[9] Berg G 2009 Plant–microbe interactions promoting plant growth and health: perspectives for controlled use of microorganisms in agriculture. Appl Microbiol Biotechnol 84:11–18. doi:10.1007/s00253-009-2092-7. 19568745

[10] Santoyo G, del Carmen Orozco-Mosqueda M, Govindappa M 2012 Mechanisms of biocontrol and plant growth-promoting activity in soil bacterial species of *Bacillus* and *Pseudomonas*: a review. Biocontrol Sci Technol 22:855–872. doi:10.1080/09583157.2012.694413.

[11] Deng X, den Bakker HC, Hendriksen RS 2016 Genomic epidemiology: whole-genome-sequencing–powered surveillance and outbreak investigation of foodborne bacterial pathogens. Annu Rev Food Sci Technol 7:353–374. doi:10.1146/annurev-food-041715-033259. 26772415

[12] Hyatt D, Chen G-L, LoCascio PF, Land ML, Larimer FW, Hauser LJ 2010 Prodigal: prokaryotic gene recognition and translation initiation site identification. BMC Bioinformatics 11:119. doi:10.1186/1471-2105-11-119. 20211023 PMC2848648

[13] Pruitt KD, Tatusova T, Maglott DR 2007 NCBI reference sequences (RefSeq): a curated non-redundant sequence database of genomes, transcripts and proteins. Nucleic Acids Res 35:D61–D65. doi:10.1093/nar/gkl842. 17130148 PMC1716718

[14] Quevillon E, Silventoinen V, Pillai S, Harte N, Mulder N, Apweiler R, Lopez R 2005 InterProScan: protein domains identifier. Nucleic Acids Res 33:W116–W120. doi:10.1093/nar/gki442. 15980438 PMC1160203

[15] Subramanian S, Smith DL 2015 Bacteriocins from the rhizosphere microbiome—from an agriculture perspective. Front Plant Sci 6:909. doi:10.3389/fpls.2015.00909. 26579159 PMC4626563

[16] Neeraja C, Anil K, Purushotham P, Suma K, Sarma P, Moerschbacher BM, Podile AR 2010 Biotechnological approaches to develop bacterial chitinases as a bioshield against fungal diseases of plants. Crit Rev Biotechnol 30:231–241. doi:10.3109/07388551.2010.487258. 20572789

[17] Pliego C, Ramos C, de Vicente A, Cazorla FM 2010 Screening for candidate bacterial biocontrol agents against soilborne fungal plant pathogens. Plant Soil 340:505–520. doi:10.1007/s11104-010-0615-8.

[18] Atanasova L, Dubey M, Grujić M, Gudmundsson M, Lorenz C, Sandgren M, Kubicek CP, Jensen DF, Karlsson M 2018 Evolution and functional characterization of pectate lyase PEL12, a member of a highly expanded *Clonostachys rosea* polysaccharide lyase 1 family. BMC Microbiol 18:178. doi:10.1186/s12866-018-1310-9. 30404596 PMC6223089

[19] Wagner A, Norris S, Chatterjee P, Morris PF, Wildschutte H 2018 Aquatic pseudomonads inhibit oomycete plant pathogens of Glycine max. Front Microbiol 9:1007. doi:10.3389/fmicb.2018.01007. 29896163 PMC5986895

[20] Matthijs S, Tehrani KA, Laus G, Jackson RW, Cooper RM, Cornelis P 2007 Thioquinolobactin, a *Pseudomonas* siderophore with antifungal and anti-*Pythium* activity. Environ Microbiol 9:425–434. doi:10.1111/j.1462-2920.2006.01154.x. 17222140

[21] Staunton J, Weissman KJ 2001 Polyketide biosynthesis: a millennium review. Nat Prod Rep 18:380–416. doi:10.1039/a909079g. 11548049

[22] Guttenberger N, Blankenfeldt W, Breinbauer R 2017 Recent developments in the isolation, biological function, biosynthesis, and synthesis of phenazine natural products. Bioorg Med Chem 25:6149–6166. doi:10.1016/j.bmc.2017.01.002. 28094222

[23] Dutkiewicz J, Mackiewicz B, Lemieszek MK, Golec M, Milanowski J 2016 *Pantoea agglomerans*: a mysterious bacterium of evil and good. Part IV. Beneficial effects. Ann Agric Environ Med 23:206–222. doi:10.5604/12321966.1203879. 27294621

[24] Scott SR, Hasty J 2016 Quorum sensing communication modules for microbial consortia. ACS Synth Biol 5:969–977. doi:10.1021/acssynbio.5b00286. 27172092 PMC5603278

[25] Pei D, Zhu J 2004 Mechanism of action of S-ribosylhomocysteinase (LuxS). Curr Opin Chem Biol 8:492–497. doi:10.1016/j.cbpa.2004.08.003. 15450491

[26] Aktuganov GE, Melent’ev AI, Galimzyanova NF, Shirokov AV 2008 The study of mycolytic properties of aerobic spore-forming bacteria producing extracellular chitinases. Microbiology 77:700–709. doi:10.1134/S0026261708060088. 19137718

[27] Daud NS, Mohd Din ARJ, Rosli MA, Azam ZM, Othman NZ, Sarmidi MR 2019 *Paenibacillus polymyxa* bioactive compounds for agricultural and biotechnological applications. Biocatal Agric Biotechnol 18:101092. doi:10.1016/j.bcab.2019.101092.

[28] Grady EN, MacDonald J, Liu L, Richman A, Yuan Z-C 2016 Current knowledge and perspectives of *Paenibacillus*: a review. Microb Cell Fact 15:203. doi:10.1186/s12934-016-0603-7. 27905924 PMC5134293

[29] Behera BC, Sethi BK, Mishra RR, Dutta SK, Thatoi HN 2017 Microbial cellulases—diversity & biotechnology with reference to mangrove environment: a review. J Genet Eng Biotechnol 15:197–210. doi:10.1016/j.jgeb.2016.12.001. 30647656 PMC6296582

[30] Yu WQ, Zheng GP, Qiu DW, Yan FC, Liu WZ, Liu WX 2019 *Paenibacillus terrae* NK3-4: a potential biocontrol agent that produces β-1,3-glucanase. Biol Control 129:92–101. doi:10.1016/j.biocontrol.2018.09.019.

